# Cancer Epigenetics: New Therapies and New Challenges

**DOI:** 10.1155/2013/529312

**Published:** 2013-02-26

**Authors:** Eleftheria Hatzimichael, Tim Crook

**Affiliations:** ^1^Department of Haematology, University Hospital of Ioannina, St. Niarchou Avenue, 45110 Ioannina, Greece; ^2^Computational Medicine Center, Jefferson Medical College, Thomas Jefferson University, Philadelphia, PA 19107, USA; ^3^Division of Cancer Research, Medical Research Institute, University of Dundee, Hospital and Medical School, Dundee DD1 9SY, UK

## Abstract

Cancer is nowadays considered to be both a genetic and an epigenetic disease. The most well studied epigenetic modification in humans is DNA methylation; however it becomes increasingly acknowledged that DNA methylation does not work alone, but rather is linked to other modifications, such as histone modifications. Epigenetic abnormalities are reversible and as a result novel therapies that work by reversing epigenetic effects are being increasingly explored. The biggest clinical impact of epigenetic modifying agents in neoplastic disorders thus far has been in haematological malignancies, and the efficacy of DNMT inhibitors and HDAC inhibitors in blood cancers clearly attests to the principle that therapeutic modification of the cancer cell epigenome can produce clinical benefit. This paper will discuss the most well studied epigenetic modifications and how these are linked to cancer, will give a brief overview of the clinical use of epigenetics as biomarkers, and will focus in more detail on epigenetic drugs and their use in solid and blood cancers.

## 1. Introduction

It has been thirty years since the “war on cancer” was declared, yet in 2008, the most recent year for which incidence and mortality rates are available, almost 12.7 million people were diagnosed with cancer and more than 7.5 million died of the disease [1]. Enormous progress has been made in the understanding of the molecular basis of carcinogenesis and the complete sequencing of the human genome represents a milestone in this quest [2]. The situation though is far more complex than a simple catalogue of genes and despite this progress the discovery of anticancer drugs remains a highly challenging endeavor and cancer a hard-to-cure disease. 

Traditionally, the development of cancer is thought to be largely due to the accumulation of genetic defects such as mutations, amplifications, deletions, and translocations affecting the cancer cell machinery and providing the cancer cell with the advantage to survive and metastasize. In addition, interactions between cancer cells and their microenvironment further support these processes [3]. Of equal importance is a second system that cells use to determine when and where a particular gene will be expressed during development. This system is overlaid on DNA in the form of epigenetic marks that are heritable during cell division but do not alter the DNA sequence [4]. The pattern of these chemical tags is called the epigenome of the cell, whereas epigenetics is the study of these marks that lead to changes in gene expression in the absence of corresponding structural changes in the genome. It is now well recognized that tumorigenesis is a multistep process involving multiple genetic and epigenetic alterations, with the latter often termed epimutations that contribute to the progressive transformation of normal cells towards a malignant phenotype, so that cancer is nowadays consider to be both a *genetic* and an *epigenetic* disease [5, 6]. Epigenetic abnormalities are reversible and as a result novel therapies that work by reversing epigenetic effects are being increasingly explored. More recently, increasing evidence suggests that genetic and epigenetic mechanisms intertwine and take advantage of each other during malignant transformation.

There are many chemical modifications that affect not only DNA, but also RNA and proteins, and create different epigenetic layers. The most well studied epigenetic modification in humans is DNA methylation; however, it becomes increasingly acknowledged that DNA methylation does not work alone, but rather is linked to other modifications, such as histone modifications. This paper will discuss the most well studied epigenetic modifications and how these are linked to cancer, give a brief overview of the clinical use of epigenetics as biomarkers, and focus in more detail on epigenetic drugs and their use in solid and blood cancers.

## 2. DNA Methylation

DNA methylation consists of the addition of a methyl group to carbon 5 of the cytosine within the dinucleotide CpG. Regions of DNA in the human genome, ranging from 0.5 to 5 kb, that are CG rich are called CpG islands and are usually found in the promoters of genes. Approximately half of all gene promoters have CpG islands that when methylated lead to transcriptional silencing. *De nov*ο** DNA methylation is brought about by DNA methyltransferases (DNMT) 3A and 3B that convert cytosine residues of CpG dinucleotides into 5-methylcytosine, whereas DNA methylation is maintained by DNMT1. 5-methylcytosine can be further converted into 5-hydroxymethyl-2′-deoxycytidine by the Ten-Eleven-Translocation (TET) family enzymes [7]. The function and significance of 5-hydroxymethylation are still unclear and under investigation. Although methylation of DNA in 5′ promoters has been well studied and has been shown to suppress gene expression, recently DNA methylation was described downstream of the promoters in intra- and inter-genic regions [8] as well in CpG shores, that is regions with lower CpG density neighboring CpG islands [9]. 

## 3. Histone Modifications

Histones are proteins around which DNA winds to form nucleosomes. A nucleosome is the basic unit of DNA packaging within the nucleus and consists of 147 base pairs of genomic DNA wrapped twice around a highly conserved histone octamer, consisting of 2 copies each of the core histones H2A, H2B, H3, and H4. Histones, however, are not only packaging elements, but also critical regulators of gene expression. Histone tails may undergo many posttranslational chemical modifications, such as acetylation, methylation, phosphorylation, ubiquitylation, and sumoylation that constitute a code, named the “histone code.” These modifications can alter the chromatin structure, from an open to a closed, condensed form and vice versa. Histone modifications act, except for chromatin packaging, on various other biological processes including transcriptional repression, gene activation, and DNA repair [10]. Three classes of histone interacting proteins have been described thus far, based on their function: the *writers *that place histone modifications, the *erasers* that can remove these modifications, and finally the *readers* that recognize the histone modifications and can deliver nucleosome, histone, or DNA-modifying enzymes.

### 3.1. Histone Acetylation

Histone acetylation occurs at either arginine-(R) or lysine-(K) residues and is a dynamic and reversible process that is regulated by two enzyme families, histone acetyltransferases (HAT) and histone deacetylases (HDAC). HATs catalyse the transfer of an acetyl group to the *ε*-amino group of the lysine residue on the histone protein and use acetyl-CoA as a cofactor. As a result chromatin adopts a more relaxed form (euchromatin) allowing the recruitment of transcription factors. HDACs reverse the acetylation of lysine residues and the local chromatin architecture becomes condensed (heterochromatin). Acetylation of lysine 16 of histone 4 (H4K16) appears to be crucial in chromatin folding and in the switch from the euchromatin state to heterochromatin [11]. Histone acetylation can also promote transcription by providing binding sites to proteins that are involved in gene activation, such as the bromodomain-containing family of proteins [12]. 

### 3.2. Histone Methylation

Histones can also be methylated at their lysine-(K) and arginine-(R) residues. Lysine residues can be monomethylated, dimethylated, or trimethylated whereas arginine residues can be mono- or dimethylated. Methyl marks are written by S-adenosylmethionine (SAM)- dependent methyltransferases and erased by either the Jumonji family of demethylases [13] or the lysine-specific histone demethylases 1 (LSD1) and 2 (LSD2) [14]. Histone methylation at lysine and arginine residues does not alter the chromatic structure, but rather acts as binding sites for other proteins that may condense chromatin [15] or have other effects. The different levels of lysine methylation are recognized by different methyl-lysine-binding domains and may be associated with either transcription activation or repression. H3K4me3, for example promotes transcription, whereas H3K27me3 is associated with gene silencing [10]. Arginine methylation of histone proteins has recently been shown to antagonize other histone marks, further increasing the histone code complexity [16].

## 4. Cancer and Epigenetic Modifications

In cancer, a global process of genomic hypomethylation occurs mostly at DNA-repetitive regions which results in activation of genes with growth and tumour promoting functions and loss of genome stability and imprinting [17]. In contrast, there are site-specific increases in CpG methylation in areas of the genome with a high density of CpG, termed CpG islands causing transcriptional silencing of tumour suppressor genes (TSG), such as *BRCA1* [18], *hMLH1* [19], *VHL* [20], *BIK* [21], and *MGMT* [22, 23]. 

Cancer contains not only DNA methylation aberrations, but also major disruption of the histone modification landscape [24]. Histone modifiers have been shown to be targets of aberrations and/or mutations in cancer such as mutated deacetylases [25], and amplified histone methyltransferases and demethylases [26]. 

### 4.1. When Genetics Meets Epigenetics in Cancer

Deregulation of the epigenetic machinery can also occur due to activation or inactivation of the epigenetic regulatory proteins. In other words, the enzymes that maintain and modify the epigenome are themselves frequent targets for mutation and/or epimutation in neoplasia [27]; for example, DNA methyltransferases themselves have been found to be genetically altered in malignancies, such as *DNMT3A* [28] and *DNMT3B* in pancreatic and breast cancer cells [29]. Somatic *DNMT3A* mutations have been described in approximately 20% of acute myeloid leukemia (AML) patients, especially in those with an intermediate risk cytogenetic profile and although they did not affect the 5-methylcytosine content [30] they were associated with poor clinical outcome [30, 31]. How the lack of effect of *DNMT3A* mutations on 5-methylcytosine content is linked to an otherwise poor clinical outcome is not yet fully understood. It has been suggested that the R882 *DNMT3A* mutations alter functions of DNMT3A such as its ability to bind other proteins involved in transcriptional regulation and localization to chromatin regions containing methylated DNA [30]. Loss-of-function *TET2* mutations were also identified in myeloid neoplasms in 20–30% [32, 33] and have been associated with both good [34] and bad prognoses [35]. 

Genome sequencing has also revealed the presence of metabolic mutations in patients with myelodysplastic syndromes (MDS) and AML related to the isocitrate dehydrogenase (*IDH*) 1 and *IDH2* genes [36]. These mutations have been reported in approximately 30% of patients with normal karyotype AML [37, 38] and have been linked to the disruption of various processes such as bone marrow microenvironment changes and impaired differentiation suggesting a proleukemogenic effect. In an AML cohort, *IDH1 *and *IDH2* mutations were mutually exclusive with *TET2* mutations while they shared the similar epigenetic defects with the *TET2* mutants. Epigenetic profiling revealed that AML patients with *IDH1/2* mutations displayed global hypermethylation and a specific hypermethylation signature [39]. MLL is another epigenetic modifier that is commonly mutated in acute leukemias and mainly due to translocations. In normal karyotype AML cases the incidence of MLL partial tandem duplications (MLL-PTD) is up to 8% whereas in cases of trisomy 11 the incidence reaches 25% [40]. Favorable AMLs such as those with t(8; 21) are MLL-PTD negative [41]. As MLL is a H3 K4 methyltransferase, translocations that replace the methyltransferase domain affect its function and have been linked with leukaemic transformation [42]. Mutations affecting the Polycomb repressive complex (PRC) components, such as EZH2, can also affect histone modifications and have recently been reported. EZH2 is the enzymatic component of the PRC2 complex and is a H3 K27 methyltransferase. Overexpression of EZH2 has been reported in various epithelial neoplasms and several types of leukemia [43–45] and has been shown to be due to, at least in part, the loss of transcriptional repression of specific microRNAs [44]. Activating mutations of *EZH2* have been reported in B-cell lymphomas [46] whereas missense, nonsense, and frameshift mutations have been reported in various myeloid malignancies [47, 48]. In AML, 3 cases so far have been described to carry *EZH2* mutations [27].

## 5. Clinical Use of Epigenetics

At present, there are two major areas of interest in the clinical use of epigenetics, namely, biomarkers and therapeutics. We now consider these areas.

### 5.1. Cancer Biomarkers

Methylated genomic DNA has a number of properties, which make it an attractive molecule for biomarker utility. First, it is stable in biofluids such as blood, urine, and saliva. Second, in the majority of cases methylation in CpG is acquired during malignant transformation and is therefore specific to neoplasia. Third, the techniques used for detection of methylated DNA are readily amenable to automation.

Several studies have explored the methylation status of gene promoters and its association with clinical parameters in primary patient samples from patients with haematological malignancies and solid tumours. Various methodologies have been used such as methylation-specific PCR (MSP), methylation-specific restriction enzyme digestion, HpaII tiny fragment enrichment by ligation-mediated PCR (HELP), bisulphite sequencing, and pyrosequencing. Either single genes or panels of genes in microarrays were studied. In MDS and AML methylation of several genes has been reported such as *MEG3*, *SNRPN* [49], *Plk2* [50], cyclin-dependent kinase inhibitors, e-cadherin [51], and various others reviewed in [52]. In multiple myeloma, methylation of the VHL promoter has been shown to correlate with bone disease [20] and methylation of the bcl-2 interacting killer (*BIK*) promoter has been shown to predict relapsed/refractory disease [21], while methylated *FHIT* has been shown to be an independent adverse prognostic factor [53].

In a study by Shen et al. [54] a panel of 10 hypermethylated genes was identified in patients with MDS. Quantitative pyrosequencing in a large cohort showed that patients with higher levels of methylation for these genes had shorter median overall and progressive-free survival (PFS) independent of age, sex, and the International Prognostic Scoring System (IPSS). Similarly, in solid tumours numerous methylated genes have been described. A substantial body of experimental evidence exists mechanistically associating acquired chemotherapy resistance with changes in the cancer cell epigenome and a number of genes have been identified, in which increased CpG island methylation and transcriptional downregulation are associated with resistance to specific agents such *hMLH1* [55] and *Plk2 *[56] in ovarian cancer. Of note, methylation-dependent silencing of the methyl transferase *MGMT* in glioblastoma multiforme confers sensitivity to the alkylating agent temozolomide [23] but as with many such candidate biomarkers, clinical application to inform patient management is not yet routine.

The list of genes reported to be methylated in haematological neoplasms is extensive, and although several have been linked to clinical parameters and have been associated with survival or response to treatment, none of these markers has been used so far in the clinic to guide diagnosis or treatment, as opposed to gene mutations such as *NPM1* and *FLT3* that are now widely used to risk classify AML patients. 

One of the major goals of investigators in oncology is that of individualized cancer therapy. Investigators continue to identify genes whose transcriptional silencing affects sensitivity to chemotherapeutic agents. The challenge now is to translate these findings into clinically usable tests to inform optimal deployment of anticancer drugs. It remains unlikely that a single gene methylation test will be sufficiently informative to guide individual patient management and it is more likely that panels of genes will be required. 

### 5.2. Cancer Therapeutics

Both epigenetic proteins and protein markers are good targets for the development of new anticancer treatments. The proof-of-concept for epigenetic therapies is the FDA and EMEA approval of demethylating agents and histone acetylase (HDAC) inhibitors for the treatment of MDS, AML and certain types of lymphomas, respectively. However, we should not forget that these agents are nonselective and their side effects are not clearly known. 

#### 5.2.1. DNA Methyltransferase Inhibitors (DNMTis) or Demethylating Agents

The two most well studied and in clinical use DNA methyltransferase inhibitors (DNMTi) are the azanucleosides azacytidine (5-azacytidine) and decitabine (5-aza′-2-deoxycytidine). Both are approved for use in the myelodysplastic syndromes and low-blast count AML and have improved the survival of patients with these diseases [57].

Unfortunately, clinical trials with DNMTi in solid tumours did not have the same results. A phase 1 study of decitabine with interleukin-2 in melanoma and renal cell carcinoma showed that decitabine caused grade 4 neutropenia in most patients [58]. Myelosuppression was also the predominant toxicity observed in a study combining decitabine with carboplatin [59]. However, in a phase II trial low-dose decitabine was found to restore sensitivity to carboplatin in patients with heavily pretreated ovarian cancer resulting in a high response rate (RR) and prolonged PFS [60]. In both studies, there was evidence that decitabine induced dose-dependent demethylation in marker genes such as *MLH1, RASSF1A, HOXA10*, and *HOXA11* [60]. It is possible that such an approach could efficiently be coupled with the use of epigenetic biomarkers predictive of chemosensitivity [56]. 

A major likely reason for the disappointing activity of demethylating agents in solid tumours is limited incorporation into cells, which are proliferating relatively slowly. These limitations may be less relevant for newer DNMTis which are independent of replication for incorporation into DNA. A second explanation for these results is that agents such as azacytidine, which cause global hypomethylation, likely reactivate expression of multiple silenced genes including oncogenes and tumour suppressors in different cell types and in different cancers. Demethylation could therefore cause both therapeutic and deleterious effects. For example, the oncogene *NT5E* is overexpressed in aggressive metastatic melanomas, yet transcriptionally silenced by methylation in breast cancer with more favorable prognosis [61]. 

A third and key possible explanation why DNMTi have advanced less rapidly in the clinic in solid tumours than in haematological malignancies is that of toxicity. Both decitabine and azacytidine are active in haematological malignancy at lower (less toxic) doses than are required for demethylation in epithelial malignancies. It is clearly of interest, therefore, that transient exposure of cells to low (relatively non-toxic) doses of these agents could induce a “memory” response with sustained reduction in CpG island methylation and reactivation of expression of previously silenced genes [62]. These observations imply that low-dose decitabine and azacytidine may have wider uses in management of neoplastic disease than previously believed. In a recently reported phase II trial Matei et al. [60] showed that pretreatment with low-dose azacytidine restored sensitivity to carboplatin in patients with drug resistant epithelial ovarian cancer and resulted in a high response rate and significantly improved clinical outcomes. This study clearly attests to the utility of low-dose azacytidine in solid tumours and sets the scene for further studies.

Newer azanucleosides are zebularine, S-110, and SGI-1027 that have shown antiproliferative activity in cell lines [63, 64], but have not entered the clinical trial setting yet.

#### 5.2.2. Histone Deacetylase Inhibitors (HDACi)

The HDACs catalyse removal of acetyl groups from lysine residues in the histones and functionally are transcriptional repressors. HDACs are divided into five classes: class I comprises HDAC1, HDAC2, HDAC3, and HDAC8; class IIa comprises HDAC4, HDAC5, HDAC7, and HDAC9; class IIb contains HDAC6 and HDAC10; class III comprises the sirtuins SIRT1-SIRT7 while class IV contains only HDAC11 [65]. The discovery of HDACi actually preceded the discovery of HDACs. Sodium butyrate was the first HDACi described to induce acetylation [66], and later on trichostatin (TSA), a fungal antibiotic, currently used in *in vitro* experiments, and valproic acid, a widely used antiepileptic, were identified. Valproic acid, in particular, has been used in combination with DNMTi and/or chemotherapy in patients with haematological malignancies [67, 68].

Currently HDACi that have been developed focus on class I and class II HDACs and can be further distinguished into chemically distinct subgroups based on their structure: aliphatic acids (phenylbutyrate, valproic acid), benzamides (entinostat), cyclic peptides (romidepsin), and hydroxamates (TSA, vorinostat/SAHA). Several HDACi are currently being tested in phase II-III trials, while two of them, vorinostat and romidepsin are the first FDA and EMEA approved agents for the treatment of progressive or recurrent cutaneous T cell lymphoma (CTCL) as second lines of treatment in 2006 and 2009, respectively [69], but convincing clinical evidence of activity of these agents in other cancer types is still lacking [70]. In non-small-cell lung cancer a number of HDACi such as entinostat, vorinostat, Pivanex, and CI-994 are in early phases of clinical development and first results have been reported [70, 71]. However, it appears that HDACi may need rational combinations to counterbalance the inherent potential of these compounds to reactivate tumor-progression genes [72]. Newer compounds such as givinostat (ITF2357) have also been developed. Givinostat has been shown to selectively target cells harboring the JAK2 V617F mutation [73] and has been tested in combination with hydroxyurea in patients with polycythemia vera in a phase II study (NCT00928707). Panobinostat (LBH589) has shown activity as monotherapy in patients with Hodgkin's lymphoma, who relapsed or were refractory to autologous transplantation [74] but limited activity in MDS [75]. However, in solid tumors the results of panobinostat monotherapy or in combination with other agents were rather disappointing [76, 77]. Second generation HDACi, such as ACY-1215, are more selective and have recently entered the clinical trial setting [78]. It would be really interesting to see the efficacy and safety profile of such compounds.

HDACi, however, do not deacetylate histones only. It becomes increasingly recognized that HDACi deacetylase other nonhistone proteins that are transcription factors, signal transducers, or even the products of oncogenes or TSG that are involved in oncogenesis [79]. This could partly explain the unacceptable toxicity [80] as well as the lack of efficacy of some compounds [81]. 

#### 5.2.3. Combination of DNMTi and HDACi

The recognition that a subset of TSGs are silenced by a combination of CpG hypermethylation and histone hypoacetylation has prompted testing of combinations of the two classes of agents and trials of these are in progress. There is initial evidence to suggest that such combinations may greatly increase clinical efficacy without unacceptable toxicity. For example, in multiply pretreated metastatic non-small-cell lung cancer patients, the combination of azacytidine and the histone deacetylase inhibitor entinostat produced objective clinical responses and, importantly, four of 19 treated patients had therapeutic responses to further agents given immediately after epigenetic therapy [82]. Evidence that demethylation is key to the responses was shown by analysis from peripheral blood samples of a set of four marker genes. The therapy was well tolerated. These encouraging results are currently being extended in further studies. The combination of decitabine and pegylated interferon alfa-2b was tested in patients with unresectable or metastatic solid tumours (NCT00701298). In ongoing trials, the combination of azacytidine and entinostat is undergoing testing in resected stage I non-small-cell lung cancer (NCT01207726) and oral azacytidine in combination with carboplatin or Abraxane (nanoparticle paclitaxel) is being evaluated in patients with refractory solid tumours (NCT01478685). 

In elderly previously untreated AML patients and high-risk MDS patients the combination of azacytidine and lenalidomide, an immunomodulator drug, is currently under investigation (NCT01442714). Both drugs as monotherapies have already shown efficacy in this group of patients so their combination seems very promising. Sequential treatment of azacytidine and lenalidomide in elderly patients with AML also showed encouraging clinical and biologic activity [83].

In a recent Phase I study decitabine was combined with bortezomib for the treatment of elderly poor risk AML patients and the combination showed good preliminary activity since response rates were very encouraging [84].

## 6. Future Promise: Therapeutics

The use of epidrugs on the intent to restore sensitivity to cytotoxic or hormonal drugs is a major goal in the setting of solid tumors [85–87]. Restoring hormonal sensitivity in breast cancer is of uppermost clinical importance and has been intensively studied over the last decades. In total 25% of breast cancers have the estrogen receptor-alpha (ER alpha) repressed mainly due to hypermethylation of the ER promoter and do not respond to endocrine therapy, and almost all hormone-sensitive tumors turn to be refractory at some point. It appears now that epigenetic therapy seems to offer a promising tool to restore/reverse hormonal sensitivity. Recent studies found that decitabine and histone HDACi such as trichostatin A, entinostat, and scriptaid can restore expression of ER mRNA and functional protein and aromatase, along with the enzymatic activity of aromatase, indicating a potential to restore long term responsiveness of a subset of ER-negative tumors to endocrine therapy [87–89].

Given the complexity and heterogeneity of the cancer cell epigenome, it is highly likely that some form of epigenomic profiling of individual cancers will be required to inform optimal use of the available agents, which induce modification of the cancer cell epigenome. For example, it would clearly be important to determine the epigenome of chemotherapy resistant cancer cells, to identify potentially deleterious silenced genes, before deploying epigenetic therapeutic strategies in an attempt to pharmacologically reverse resistance. Malignant melanoma is an interesting example of such an approach. In this tumor type, loss of 5-hydroxymethylcytosine (5-hmC) has diagnostic and prognostic implications, which relates to downregulation of IDH2 and TET family enzymes. Reintroducing active TET2 or IDH2 was found to suppress melanoma growth and increase tumor-free survival in animal models [90].

Identifying the epigenetically modified genes, which are principally involved in tumor resistance, can be achieved by comparative analysis of diagnostic (pretreatment) biopsy with a second biopsy at disease relapse. Such rebiopsying is rapidly becoming the standard of care in oncology, for example, in breast cancer [91]. 

The ability of the physician to exploit therapeutic opportunities created by epigenetic changes in the cancer cell epigenome may also offer new approaches to cancer management. For example, *ASS1*, which encodes arginine succinate synthetase, the rate-limiting enzyme in arginine biosynthesis, is silenced by methylation in some cancer types including renal cell carcinoma, hepatocellular carcinoma, malignant melanoma, glioblastoma multiforme (GBM), and platinum-resistant epithelial ovarian cancer. *ASL* encoding arginine succinate lyase (a second key enzyme in arginine biosynthesis) is also silenced by CpG island methylation in GBM [92]. Loss of either gene confers arginine auxotrophy and sensitivity to arginine deiminase. These observations imply a further form of epigenetic therapy in which biochemical abnormalities resulting from epigenetic changes can be targeted for clinical benefit. 

As we previously discussed, several epigenetic modifiers such as EZH2, IDH1/2, and DNMT3A are genetically altered in cancer. These epigenetic modifiers provide now new therapeutic targets for clinical development. What seems to be needed though is a better selection of patients who will benefit from such treatments as well as identification of new druggable targets and compounds such as histone kinases [93] or inhibitors of histone methyltransferases [94] and sirtuins [95]. 

## 7. Conclusions

The biggest clinical impact of epigenetic modifying agents in neoplastic disorders thus far has been in haematological malignancies and the efficacy of DNMTis and HDACi in blood cancers clearly attests to the principle that therapeutic modification of the cancer cell epigenome can produce clinical benefit. Although the efficacy of epigenetic therapy in solid tumours remains as yet unproven, there is every reason to believe that more rational use of existing agents, perhaps informed by individual patient epigenetic profiling, will improve the therapeutic index of this approach. Furthermore, an increasing number of viable new therapeutic targets are emerging from increased understanding of the epigenetic regulatory circuitry and its derangement in neoplasia. 
